# Emergency Department Pain Management Following Implementation of a Geriatric Hip Fracture Program

**DOI:** 10.5811/westjem.2017.3.32853

**Published:** 2017-04-19

**Authors:** Scott D. Casey, Dane E. Stevenson, Bryn E. Mumma, Christina Slee, Philip R. Wolinsky, Calvin H. Hirsch, Katren Tyler

**Affiliations:** * University of California, Davis School of Medicine, Department of Emergency Medicine, Sacramento, California; † UC Davis Medical Center, Department of Quality and Safety, Sacramento, California; ‡ University of California, Davis School of Medicine, Department of Orthopaedic Surgery, Sacramento, California; § University of California, Davis School of Medicine, Department of Internal Medicine, Sacramento, California

## Abstract

**Introduction:**

Over 300,000 patients in the United States sustain low-trauma fragility hip fractures annually. Multidisciplinary geriatric fracture programs (GFP) including early, multimodal pain management reduce morbidity and mortality. Our overall goal was to determine the effects of a GFP on the emergency department (ED) pain management of geriatric fragility hip fractures.

**Methods:**

We performed a retrospective study including patients age ≥65 years with fragility hip fractures two years before and two years after the implementation of the GFP. Outcomes were time to (any) first analgesic, use of acetaminophen and fascia iliaca compartment block (FICB) in the ED, and amount of opioid medication administered in the first 24 hours. We used permutation tests to evaluate differences in ED pain management following GFP implementation.

**Results:**

We studied 131 patients in the pre-GFP period and 177 patients in the post-GFP period. In the post-GFP period, more patients received FICB (6% vs. 60%; difference 54%, 95% confidence interval [CI] 45–63%; p<0.001) and acetaminophen (10% vs. 51%; difference 41%, 95% CI 32–51%; p<0.001) in the ED. Patients in the post-GFP period also had a shorter time to first analgesic (103 vs. 93 minutes; p=0.04) and received fewer morphine equivalents in the first 24 hours (15mg vs. 10mg, p<0.001) than patients in the pre-GFP period.

**Conclusion:**

Implementation of a GFP was associated with improved ED pain management for geriatric patients with fragility hip fractures. Future studies should evaluate the effects of these changes in pain management on longer-term outcomes.

## INTRODUCTION

Every year over 300,000 Americans sustain low-trauma fragility hip fractures^1^–^5^ at an estimated cost of over $12 billion.^6^ Following a hip fracture, inpatient mortality is around 4%^7^ and 12-month mortality is 20–25%.^4^,^8^ Only half of patients sustaining a hip fracture recover their pre-fracture mobility.^7^ Multidisciplinary geriatric fracture programs (GFP) reduce mortality,^9^ morbidity,^9^–^11^ and hospital costs.^12^ GFP interventions include early multimodal pain management,^13^ delirium prevention,^14^ management of medical co-morbidities,^13^,^14^ early operative fixation,^15^,^16^ early mobilization,^17^ and early discharge planning.^10^,^11^,^17^ Many GFPs also include preoperative regional anesthesia that has been shown to reduce overall opioid requirements,^18^ reduce rates of delirium^19^ and relieve pain more effectively than standard care.^20^ Other elements of multimodal pain management include acetaminophen,^13^ urinary catheter use,^17^ and patient positioning.

Our overall goal was to determine the effects of a multidisciplinary GFP on the emergency department (ED) pain management of fragility hip fractures. We hypothesized that the implementation of a GFP in the ED would be associated with increases in the use of regional anesthesia and acetaminophen and decreases in the time to first analgesic and amount of opioid medication.

## METHODS

### Study Design

This was a retrospective, before-and-after cohort study using data from the University of California, Davis Health System’s electronic health record (EHR). This study was approved by our institutional review board.

### Study Setting and Population

We performed this study at a single urban, academic ED with an annual volume of approximately 60,000 adult patients. Our hospital is a tertiary care facility with 619 licensed acute care beds and serves a 65,000 square-mile area that includes 33 counties and six million residents. Our hospital implemented the GFP on January 1, 2014. The GFP was developed by the departments of orthopaedics, internal medicine, anesthesiology, pharmacy and emergency medicine and was started as a quality improvement program on January 1, 2014. The program includes osteoporosis screening, medical co-management, operative fixation within 48 hours, early physical/occupational therapy including mobilization and early discharge planning, as well as strategies to recognize, prevent and manage delirium. The GFP team meets weekly to discuss patient and system issues. In the ED, the GFP includes a multimodal pain-control order set consisting of early acetaminophen, opioid medication, and fascia iliaca compartment block (FICB) regional anesthesia.^21^ ED providers received both didactic and practical training on the administration of FICB in the fall of 2013 and approximately annually thereafter. They were also educated on opioid and non-opioid strategies for pain relief. Information was also distributed via email and posters in the ED. Emergency medicine residents perform most FICBs. Indications for FICB include moderate to severe pain or receipt of two or more doses of opioids. Contraindications include, but are not limited to, use of anticoagulants or oral antiplatelet agents (not including aspirin) and inability to obtain informed consent. During both periods, the study site ED’s procedure was to complete a pain assessment (a) immediately upon presentation at hospital and (b) within 30 minutes of administering initial analgesia, and (c) regularly as part of routine nursing observations throughout ED stay. The pre-GFP period extended from December 27, 2011, to December 31, 2013, and the post-GFP period extended from January 1, 2014, to January 9, 2016.

**Population Health Research Capsule**

What do we already know about this issue?

*Geriatric* *fracture* *programs (GFP) reduce mortality, morbidity, and hospital costs for geriatric patients with hip fractures.*

What was the research question?

Does a GFP improve ED pain management for geriatric patients with hip fractures?

What was the major finding of the study?

A GFP was associated with enhanced ED pain management for geriatric patients with hip fractures.

How does this improve population health?

A GFP was associated with decreased variability in analgesia timing and use and with more patients receiving evidence-based pain management.

We included all patients age 65 years and older who presented via the ED with a unilateral, native, non-pathologic, low-energy, proximal femur fracture (including subcapital, intertrochanteric and subtrochanteric hip fractures) who were admitted to the hospital. We excluded patients under 65 years of age, fractures resulting from high-energy mechanisms (ex. motor vehicle collision, falls from greater than five feet), periprosthetic fractures, isolated trochanteric fractures, femoral shaft fractures and patients with multiple injuries.

### Study Protocol

Eligible patients were initially identified based on an International Classification of Diseases (ICD)-9-CM code of 820.xx, 821.xx, or 733.14 prior to October 1, 2015, or an equivalent ICD-10 code (Appendix A) after October 1, 2015. These charts were manually reviewed for the inclusion and exclusion criteria. The following elements were directly extracted from the EHR: sex, age, admitting service, and American Society of Anesthesiologists class. The following elements were manually abstracted from the EHR using a standardized form designed a priori: race, ethnicity, acetaminophen administration in the ED, FICB use, contraindications for FICB, and complications of FICB. Time to imaging, time to surgery, ED length of stay, time to first analgesic, time to first opioid analgesic, time to acetaminophen administration and total intravenous (IV) morphine equivalents outside of the operating room in the first 24 hours were calculated from data directly and manually abstracted from the EHR. One reviewer abstracted patient data for all outcomes. The reviewer was blinded to the study’s hypotheses and patient group (pre- vs. post-GFP period). An independent reviewer randomly selected 30 charts and abstracted data on two outcomes (IV morphine equivalents and ED acetaminophen administration). We collected and managed study data using REDCap electronic data capture tools hosted at the University of California, Davis.^22^

### Key Outcome Measures

Our primary outcomes were FICB use in the ED, acetaminophen use in the ED, time to first analgesia, and IV morphine equivalents administered in the first 24 hours. We also evaluated race and sex differences in these outcomes.

We defined ED length of stay as the time from ED triage to the time that the patient physically left the ED. ED acetaminophen was defined as any administration of acetaminophen (oral, rectal, or IV) while the patient was in the ED. Time to first analgesic was defined as the time from ED triage to first administration of any analgesic. IV morphine equivalents in the first 24 hours included all opioid medications administered outside the operating room within 24 hours of ED arrival. We used a calculator approved and used by the UC Davis Medical Center Pharmacy and Therapeutics Committee to convert all other opioid medications to IV morphine equivalents (Appendix B).

### Data Analysis

We calculated summary statistics. To evaluate inter-rater reliability, we used kappa coefficient for the binary outcome and Pearson’s correlation coefficient for the continuous outcome. Chi-square and Fisher’s exact tests were used to compare patients in the pre-GFP and post-GFP periods. We compared binary outcomes between the pre-GFP and post-GFP period using two-sample binomial Z-tests. Both time to first analgesic and IV morphine equivalents had skewed distributions. Hence, we used regression models and permutation tests to assess the statistical significance for independent variables. To compare mean differences in these outcomes between periods, we fit regression models to these outcomes and applied permutation tests to the resulting regression coefficients to obtain valid p-values.^23^ To compare differences between the pre- and post-GFP periods we fit simple regression models. To assess sex and race differences, we fit a multiple regression model that adjusted for period, race, and sex. Equality of variance was analyzed using median-based Levene testing. For all analyses, a p<0.05 was considered statistically significant. We performed analyses using Stata Version 14.1 (StataCorp LP, College Station, TX).

## RESULTS

Of 325 patients with eligible diagnosis codes, 17 patients were excluded from the study due to non-isolated injuries (6), high-energy mechanism (5), peri-prosthetic fracture (2), femur fracture with no hip involvement (2), lack of an acute fracture (1), and pathologic fracture (1). We studied 131 patients in the pre-GFP period and 177 patients in the post-GFP period. The majority of patients in the study were female (213, 69%) and White (194, 63%). Median age was 82 years. Demographic and clinical characteristics of the two groups are shown in Table 1.

The two reviewers had perfect agreement for ED acetaminophen use (30/30, 18 “yes;” kappa=1.00) and excellent agreement on outcomes for morphine equivalents (correlation coefficient 0.94). In the post-GFP period, more patients received FICB (6% vs. 60%; difference 54%, 95% CI 45–63%; p<0.001) and acetaminophen (10% vs. 51%; difference 41%, 95% CI 32–51%; p<0.001) in the ED. Patients had shorter time to first analgesic (103 vs. 93 minutes; p=0.04) and received fewer morphine equivalents in the first 24 hours (15mg vs. 10mg, p<0.001). Differences in time to imaging, ED length of stay, and time to surgery were not statistically significant between the pre-GFP and post-GFP periods. (Table 2)

No cases of local anesthetic systemic toxicity or other complications were reported for patients who received FICB (0/107; 0%, 95% CI 0–3.3%). Seventy patients (70/177; 40%) in the post-GFP period did not receive FICB. Of these 70 patients, the procedure was contraindicated in 40 patients (57%) due to anticoagulation therapy, nine patients (13%) due to refusal, and one patient (1%) due to anesthetic allergy. In 20 of the 70 patients (29%) there was no documented contraindication to FICB in the EHR.

**Figure 1:**
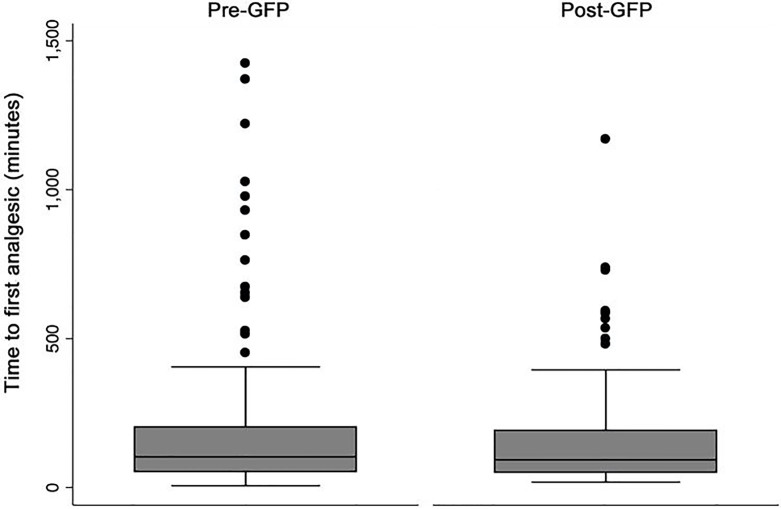
Time to first analgesic in minutes before and after implementation of a geriatric fracture program (GFP).

**Figure 2:**
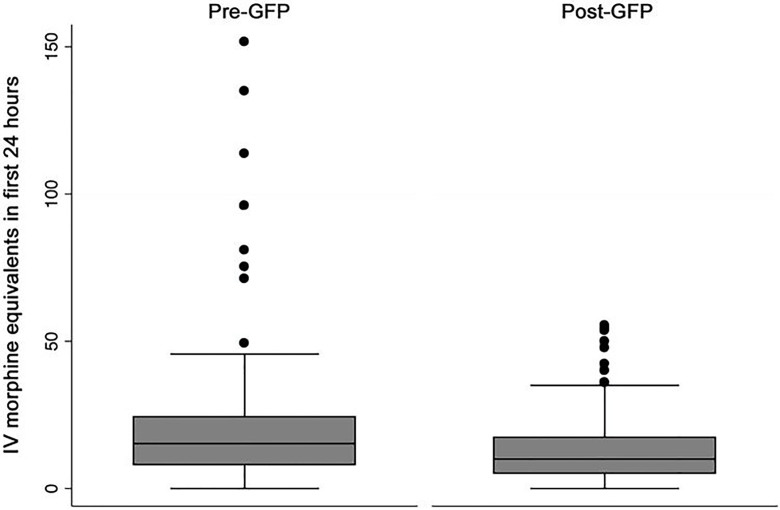
Intravenous (IV) morphine equivalents (mg) before and after implementation of a geriatric fracture program (GFP).

We observed less variance in amount of opioid medication used (p=0.006) and time to first analgesic (p=0.03) in the post-GFP period (Figures 1 and 2).

Notably, seven patients in the pre-GFP period but no patients in the post-GFP period received over 60mg IV morphine equivalents for pain control in the first 24-hour period. Twelve patients in the pre-GFP period but only three patients in the post-GFP period received their first analgesic over 600 minutes after ED arrival.

In univariable analysis, non-White patients received less opioid medication than White patients (p=0.03). This association persisted (p=0.03) in a multivariable analysis adjusting for pre- vs. post-GFP period and sex. There was no interaction between race and pre- vs. post-GFP period (p=0.07) with regard to opioid timing. No differences were found in time to first analgesic, acetaminophen use or FICB use between White and non-White patients (data not shown). No sex differences were found in any of the four outcomes (data not shown).

## DISCUSSION

We provide one of the first reports of a GFP’s effect on ED pain management of fragility hip fractures in the United States. Overall, our results suggest that patients received earlier and more comprehensive ED pain management following the implementation of a GFP as evidenced by increased usage of regional anesthesia and acetaminophen along with decreased patient opioid requirements and time to first analgesia. The decrease in opioid use was likely due to pain relief provided by the FICB and acetaminophen. Pain management in this population is important because good pain control is associated with increased mobility, fewer complications resulting from immobility, and decreased rates of delirium. Rapid pain management in this population is also important because time to administration of oral, parenteral or intranasal pain medication is a Medicare quality measure for patients presenting with a long bone fracture.^24^

Our data demonstrate the feasibility and safety of FICB performed in the ED by emergency physicians (EP). We adopted conservative guidelines from the American Society of Regional Anesthesia and Pain Medicine^25^ in the design of the FICB clinical pathway and received support from the departments of anesthesia and pharmacy. FICB was chosen over femoral nerve block to avoid injury to the vascular bundle and to decrease the risk of local anesthetic systemic toxicity. To our knowledge, no patients suffered local anesthetic systemic toxicity or other complications from the FICB. The safety profile we observed is comparable to that reported in other studies.^19^,^26^–^28^ Our data suggest that most patients are both eligible for and agreeable to FICB as pain management. Importantly, our FICB clinical pathway had no effect on ED length of stay, time to imaging or time to surgery. This result suggests that FICB can be incorporated into a patient’s ED pain management without delaying other aspects of hip fracture care. At our institution, written informed consent is required prior to FICB performance. In the post-GFP period, patients with dementia but no documented contraindications may have lacked a healthcare proxy to consent to the procedure.

The GFP’s multimodal pain management education for resident and attending physicians regarding FICB was critical to the program’s success. We held annual FICB training sessions with didactic and practical components for EPs. We discovered a need for continued re-education, particularly in July with the arrival of new EPs who were unfamiliar with the FICB clinical pathway.

We found less variability in the post-GFP period in both opioid requirements and time to first analgesia. These differences are likely multifactorial. First, the GFP included an EHR order set that included acetaminophen and set doses of opioid medication. Second, ED provider education emphasized early pain relief. Third, the use of oral or rectal acetaminophen was stressed with occasional use of IV acetaminophen. In our ED, acetaminophen can be ordered by a physician in triage and administered prior to IV access. This decreased variability suggests that more patients are receiving the evidence-based, high-level standard of care included in our GFP clinical pathway.^17^

Non-White patients received less total opioid medication than White patients; however, no racial differences existed in other ED pain management measures. The existence of racial disparities in ED opioid prescribing for long bone fracture is controversial.^29^–^32^ The reasons for this difference in our data are unclear. ED providers may have unconscious racial bias and administer less opioid medication to non-White patients.^33^,^34^ Alternatively, non-White patients may request less opioid medication due to cultural differences in pain management strategies. Further research is necessary to confirm this difference and elucidate the reasons for it. Notably, no sex differences were found in the ED pain management of this population.

## LIMITATIONS

Our study has several limitations. While we used strategies to minimize bias, our study is subject to limitations inherent in retrospective studies.^35^ We were able to show association between the GFP and outcomes, not causation. Contraindications for FICB were dependent on accurate clinical documentation. Similarly, we were unable to compare pain scores and rates of delirium between the two periods because they were not reliably documented in the pre-GFP period. We evaluated analgesia use in the first 24 hours following ED presentation; possible differences in opioid use after this period remain unknown. The GFP’s effect on several other important outcomes such as in-hospital mortality, length of hospital stay and time to ambulation following surgery remain unknown and warrant investigation.

## CONCLUSION

Implementation of a GFP was associated with improved ED pain management for geriatric patients with fragility hip fractures via ED use of FICB and acetaminophen. Future studies should evaluate the effects of these interventions on longer-term patient outcomes.

## Supplementary Information





## Figures and Tables

**Table 1:** Patient characteristics before and after implementation of a geriatric fracture program in the emergency department.

	Pre-GFP period (n=131 patients)		Post-GFP period (n=177 patients)		p-value
Age (years)	*83 (77–88)		*82 (74–88)		0.9
Female sex	93	71%	120	68%	0.5
Race					0.5
White	89	68%	125	71%	
Black	10	8%	10	6%	
Asian	13	10%	12	7%	
Other	9	7%	20	11%	
Missing	10	8%	10	6%	
Ethnicity					0.2
Hispanic	5	4%	10	6%	
Missing	11	8%	7	4%	
Admitting service					0.02
Orthopedics	88	67%	114	64%	
Internal medicine	10	8%	33	19%	
Trauma surgery	23	18%	24	14%	
Intensive care	1	1%	1	1%	
Missing	9	7%	5	3%	
ASA class					0.3
Class 1	0	0%	0	0%	
Class 2	18	14%	18	10%	
Class 3	76	58%	118	67%	
Class 4	31	24%	32	18%	
Class 5	0	0%	1	1%	
Missing	6	5%	8	5%	

*GFP*, geriatric fracture program; *ASA*, American Society of Anesthesiologists.

* Data presented as median (Q2–Q3).

**Table 2:** ED pain management and time intervals before and after implementation of a geriatric fracture program.

Clinical outcome	Pre-GFP period (n=131 patients)	Post-GFP period (n=177 patients)	p-value
Time to first pain medication (minutes)*	103 (52–203)	93 (50–192)	p=0.04
Time to first opioid medication (minutes)*	103 (52–203)	104 (51–220)	p=0.15
Morphine equivalents in first 24 hours (mg)*	15 (8–24)	10 (5–17)	p<0.001
Acetaminophen use in ED	13 (10%)	91 (51%)	p<0.001
FICB use in ED	8 (6%)	107 (60%)	p<0.001
Time to imaging (minutes)*	70 (47–137)	111 (70–167)	p=0.20
ED length of stay (hours)*	8.7 (6.4–11.7)	8.7 (7–12.5)	p=0.65
Time to surgery (hours)*	25 (19–39)	26 (20–41)	p=0.39

*GFP*, geriatric fracture program; *ED*, emergency department; *FICB*, fascia iliaca compartment block.

* Data presented as median (Q2–Q3).
