# Supplementary material for: Emergency Department Pain Management Following Implementation of a Geriatric Hip Fracture Program
Source: West J Emerg Med. 2017 Apr 19;18(4):585–91. doi: 10.5811/westjem.2017.3.32853 (PMC5468062; doi:10.5811/westjem.2017.3.32853)
Supplement: Supplementary file 1 [file wjem-18-585-s001.docx]

**Appendix A.** List of ICD-10 codes.

The following ICD-10 codes were used to identify eligible patients: M84.459A, M84.453A, S72.019A, S72.023A, S72.026A, S72.033A, S72.036A, S72.043A, S72.046A, S72.099A, S72.019B, S72.019C, S72.023B, S72.023C, S72.026B, S72.026C, S72.033B, S72.033C, S72.036B, S72.036C, S72.043B, S72.043C, S72.046B, S72.046C, S72.099B, S72.099C, S72.109A, S72.143A, S72.146A, S72.23XA, S72.26XA, S72.109B, S72.109C, S72.143B, S72.143C, S72.146B, S72.146C, S72.23XB, S72.23XC, S72.26XB, S72.26XC, S72.009A, S72.009B, S72.009C, S72.90XA, S72.309A, S72.90XB, S72.90XC, S72.309B, S72.309C, S72.409A, S72.413A, S72.416A, S72.443A, S72.446A, S72.453A, S72.456A, S72.499A, S72.409B, S72.409C, S72.413B, S72.413C, S72.416B, S72.416C, S72.443B, S72.443C, S72.446B, S72.446C, S72.453B, S72.453C, S72.456B, S72.456C, S72.499B and S72.499C.
