# Supplementary material for: Emergency Department Pain Management Following Implementation of a Geriatric Hip Fracture Program
Source: West J Emerg Med. 2017 Apr 19;18(4):585–91. doi: 10.5811/westjem.2017.3.32853 (PMC5468062; doi:10.5811/westjem.2017.3.32853)

**Appendix B**. Calculation factors for IV morphine equivalents.

| Medication | Dose | IV Morphine Equivalents |  |  |  |  |  |
| --- | --- | --- | --- | --- | --- | --- | --- |
| Intravenous |  |  |  |  |  |  |  |
| Fentanyl | 1 mcg | 0.1 |  |  |  |  |  |
| Hydromorphone | 1 mg | 6.7 |  |  |  |  |  |
| Morphine | 1 mg | 1.0 |  |  |  |  |  |
| Oral |  |  |  |  |  |  |  |
| Hydrocodone | 1 mg | 0.3 |  |  |  |  |  |
| Morphine | 1 mg | 0.3 |  |  |  |  |  |
| Oxycodone | 1 mg | 0.5 |  |  |  |  |  |
| IV = Intravenous |  |  |  |  |  |  |  |

-----------------------------------------------------------------------------------------------------------

**Appendix B Table a bit confusing – IV Morphine Equivalents – IF….**

**TOP Section**

**IV Equivalents – based on 1 mg Morphine – the Hydromorphone =0.2 (not 6.7)**


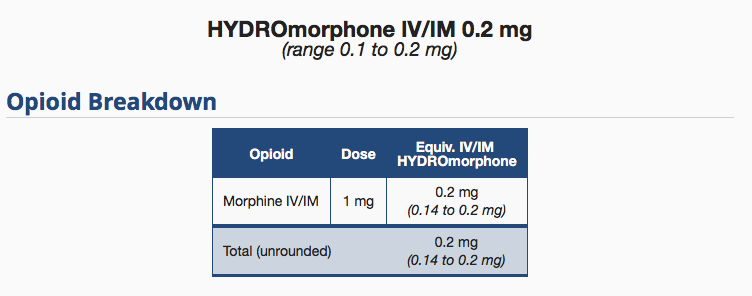


**BOTTOM Section**

**PO Equivalents – based on 0.3 mg Morphine the Oxycodone = 0.15mg (not 0.5)**


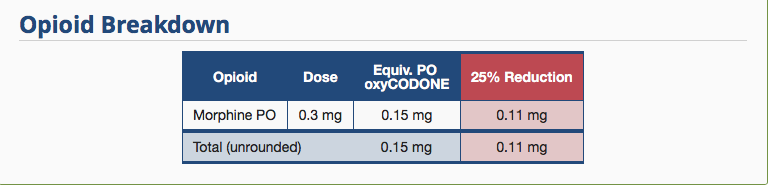

Supplement: Supplementary file 2 [file wjem-18-585-s002.docx]
